# Host-pathogen reorganisation during host cell entry by *Chlamydia trachomatis*

**DOI:** 10.1016/j.micinf.2015.08.004

**Published:** 2015

**Authors:** Andrea Nans, Charlotte Ford, Richard D. Hayward

**Affiliations:** Institute of Structural and Molecular Biology, University College London & Birkbeck, Malet Street, London WC1E 7HX, UK

**Keywords:** Chlamydia, Type III secretion, Entry, Cytoskeleton, Cryo-electron tomography

## Abstract

*Chlamydia trachomatis* is obligate intracellular bacterial pathogen that remains a significant public health burden worldwide. A critical early event during infection is chlamydial entry into non-phagocytic host epithelial cells. Like other Gram-negative bacteria, *C. trachomatis* uses a type III secretion system (T3SS) to deliver virulence effector proteins into host cells. These effectors trigger bacterial uptake and promote bacterial survival and replication within the host cell. In this review, we highlight recent cryo-electron tomography that has provided striking insights into the initial interactions between *Chlamydia* and its host. We describe the polarised structure of extracellular *C. trachomatis* elementary bodies (EBs), and the supramolecular organisation of T3SS complexes on the EB surface, in addition to the changes in host and pathogen architecture that accompany bacterial internalisation and EB encapsulation into early intracellular vacuoles. Finally, we consider the implications for further understanding the mechanism of *C. trachomatis* entry and how this might relate to those of other bacteria and viruses.

## 1 Introduction

*Chlamydia trachomatis* is an obligate intracellular bacterial pathogen. *Chlamydiae* cause diseases in humans and other animals, and in particular *C. trachomatis* remains the leading bacterial cause of sexually transmitted disease worldwide [1], while ocular infections cause blinding trachoma, which is designated as a neglected tropical disease by the World Health Organisation [2].

In common with other bacterial pathogens, a critical early step in chlamydial infection is the interaction of infectious but metabolically inactive extracellular elementary bodies (EBs) with the host cell plasma membrane. Adherent EBs trigger host actin reorganisation and membrane deformation, and rapidly internalise into endocytic vacuoles. These early bacteria-containing vacuoles then coalesce and traffic to the microtubule-organising centre, where they fuse to form a single specialised membrane-bound compartment termed an inclusion that remains segregated from the host endosomal pathway. Subsequently, EBs differentiate to form metabolically active reticulate bodies (RBs), which divide by binary fission before re-differentiating into EBs. Infectious EBs are then released from the host cell by inclusion extrusion or upon cell lysis [3]. In this review, we describe recent insights into EB structure and the morphological changes in pathogen and host that accompany EB internalisation. We discuss the implications for understanding the mechanism of *C. trachomatis* entry into host cells.

*C. trachomatis* EBs are atypically small Gram-negative cocci 0.3–0.4 μm in diameter. A long-recognised distinctive structural characteristic is their outer membrane, which is twice the normal thickness [4]. This is most likely due to the disulphide cross-linked network of major outer membrane proteins that confer the osmotic stability and rigidity essential for their extracellular lifestyle [5], [6]. Both EBs and RBs possess type III secretion systems (T3SSs), envelope-spanning nanomachines conserved among diverse Gram-negative bacterial pathogens. T3SSs translocate virulence effector proteins directly into host cells, where they subvert cellular processes to promote bacterial entry, survival and replication [7]. Although it is not possible to selectively mutate T3SS-associated genes in *Chlamydiae*, chemical inhibition of T3SSs attenuates chlamydial entry and intracellular replication, arresting the bacterial lifecycle [8], [9]. This demonstrates the importance of T3SS effectors at multiple stages of the chlamydial developmental cycle.

## 2 The polarised architecture of *C. trachomatis* EBs

**Fig. 1:**
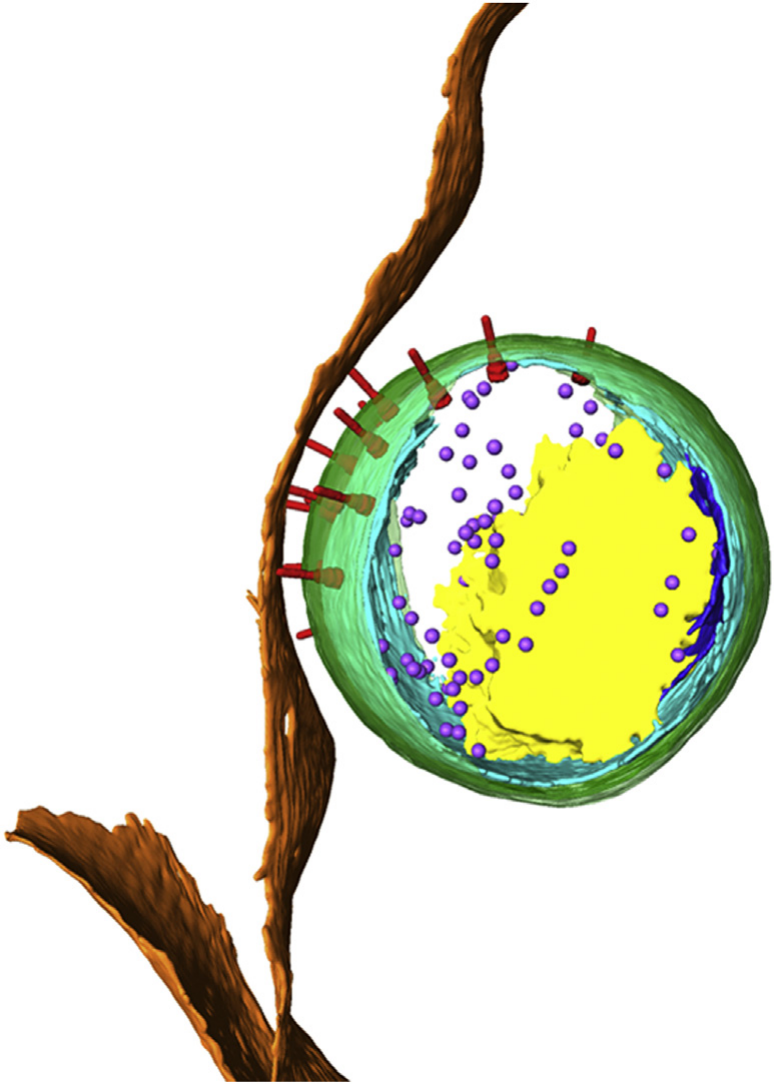
**Polarised structure of the *Chlamydia trachomatis* elementary body in contact with the host cell**. Three-dimensional surface representation of a *Chlamydia trachomatis* elementary body in contact with the host cell, generated from segmentation of a cryo-electron tomogram. Cellular plasma membrane (orange), bacterial outer membrane (green), inner membrane (cyan), inner membrane invagination (blue), T3SS (red), ribosomes (purple) and DNA nucleoid (yellow) are shown.

Early electron microscopy studies of chlamydial EBs in the absence of host cells by Matsumoto identified surface projections and surface complexes termed ‘rosettes’ [*e.g.* Ref. [10]]. Although these were later proposed to be T3SSs [11], the rosettes visualised by negative staining of the isolated *Chlamydia psittaci* envelope were also suggested to be outer membrane protein complexes [12]. Recently we have applied cryo-electron tomography to examine the structure of EBs in greater detail, both in isolation and during their entry into host cells [13]. This revealed that EBs have an inherently polarised architecture (Fig. 1). One bacterial hemisphere is characterised by a pronounced widening of the periplasmic space (∼29 nm compared to ∼14 nm on the opposite pole) that accommodates a semi-ordered array of 14–20 trans-periplasmic complexes with an average spacing of 56.5 nm ± 1.0 nm. Each complex originates at a distinct concave deformation of the inner membrane and contains a short ∼30 nm needle-like filament that protrudes from the rigid bacterial outer membrane. The overall size and shape of these complexes are consistent with the T3SS, and labelling of the *Chlamydia* T3SS needle-forming protein (CdsF) [14] by immuno-gold electron microscopy demonstrated a similarly polarised distribution, confirming these complexes as T3SSs for the first time [13]. To date, this polarised battery of T3SSs is unique to *Chlamydia*, as other Gram-negative bacterial pathogens typically distribute their T3SSs evenly around the entire bacterial surface [*e.g.* Ref. [15]]. This specialised localisation might permit *Chlamydia* to concentrate the delivery of translocated effectors into the host cell cytosol, potentially enhancing the speed and efficacy of downstream effects such as actin polymerisation, membrane deformation, or the subversion of other host signalling pathways central to its intracellular lifestyle.

The opposite pole with a narrower periplasmic space contains additional complexes of distinct morphology but unknown composition. These comprise trans-periplasmic bands of density with an average spacing of 14.5 nm ± 2.8 nm. In addition, an invagination of the inner membrane is present. This is an elongated membrane tubule in the native state, and can adopt a spherical topology after EBs are stressed by freeze-thaw, indicating a degree of morphological plasticity. In both states this is a significant structure, as the surface area is equivalent to 10–12% of the total inner membrane [13]. Although its function remains unknown, the invagination is perhaps reminiscent of the complex organelle-like membrane structures present in other members of the *Planctomycetes-Verrucomicrobia-Chlamydiae* [16].

## 3 EB-host interactions during early stages of cell entry

When visualised in the presence of host cells, all the EBs including those not directly adjacent to a host cell, oriented their T3SS array towards the host plasma membrane [13]. Whether this positioning and ensuing T3SS needle contact requires additional engagement of host receptors or polysaccharides implicated in chlamydial adhesion remains to be determined, although it is tempting to speculate that bacterial outer membrane adhesins such as OmcB and the family of polymorphic membrane proteins (Pmps) might also be polarised on the EB surface [17], [18]. Strikingly, needles of the T3SS were frequently captured in direct contact with the host plasma membrane, providing a first view of the initial events that occur during effector translocation (Fig. 1) [13].

**Fig. 2:**
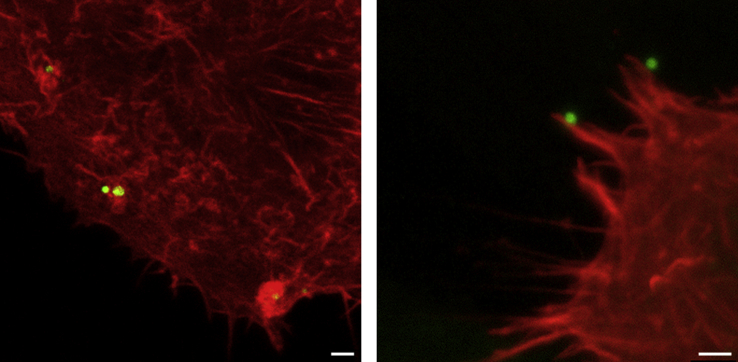
**Diverse interactions between EBs and host cells**. Confocal micrographs of cultured RPE1 cells expressing LifeAct to visualise actin filaments (red) 30 min after infection with *C. trachomatis* LGV2 labelled with AlexaFluor 488 (green). Left panel shows membrane ruffles, right panel shows filopodial capture. Scale bars, 1 μm.

Our cryo-electron tomography also captured an unexpected diversity of early entry structures including phagocytic cups that tightly zipper around individual EBs. Distinct loops of membrane, from which actin filaments emanate, pinch away from these phagocytic cups, potentially providing one of the driving forces necessary for EB internalisation [13]. *C. trachomatis* induce microvilli at entry sites, which have been observed by live fluorescence microscopy and scanning electron microscopy [19]. Correspondingly, EBs were frequently observed attached to and trapped at the base of filopodia by tomography [13]. In addition to these more defined structures, complex membrane ruffles and macropinosomes were also involved in the engulfment of *C. trachomatis* EBs. Although actin filaments accumulated at entry sites, the degree of actin recruitment was unexpectedly not as extensive as that in membrane ruffles generated by T3SS effectors delivered by *Salmonella* during host cell entry [20], suggesting some mechanistic divergence. However, it is not possible to assess by electron tomography alone whether these host-pathogen structures represent sequential assemblies in a single pathway or reflect multiple independent entry mechanisms. Detailed live fluorescence microscopy and cell biology approaches are now required to resolve a much larger number of entry events to address this question and to define the participating signalling pathways (Fig. 2).

Although morphologically diverse, the induced membrane invaginations initially remain open to the fluid phase and frequently contain multiple EBs. Subsequently, these structures close to capture a single EB in a loose vacuole together with host membrane fragments and material from the extracellular milieu. More often, tight bacterial-containing vacuoles, with a minimal lumen in which the vacuolar membrane tracks the contour of the encapsulated EB are also observed, suggesting that sorting and reorganisation of the host membranes actively continues in the first few hours post entry. These transitions in vacuole architecture are accompanied by associated changes in bacterial structure. Internalised EBs lose their defined polarity as there is a reduction of the characteristic periplasmic widening and a coupled decrease in the number of assembled T3SS complexes. Nevertheless, the inner membrane invagination remains present at one hemisphere, suggesting that this feature might be important for membrane expansion during EB to RB differentiation later in the lifecycle [13].

## 4 The enigmatic mechanism of *C. trachomatis* cell entry: ‘zipper’, ‘trigger’ or something else?

**Fig. 3:**
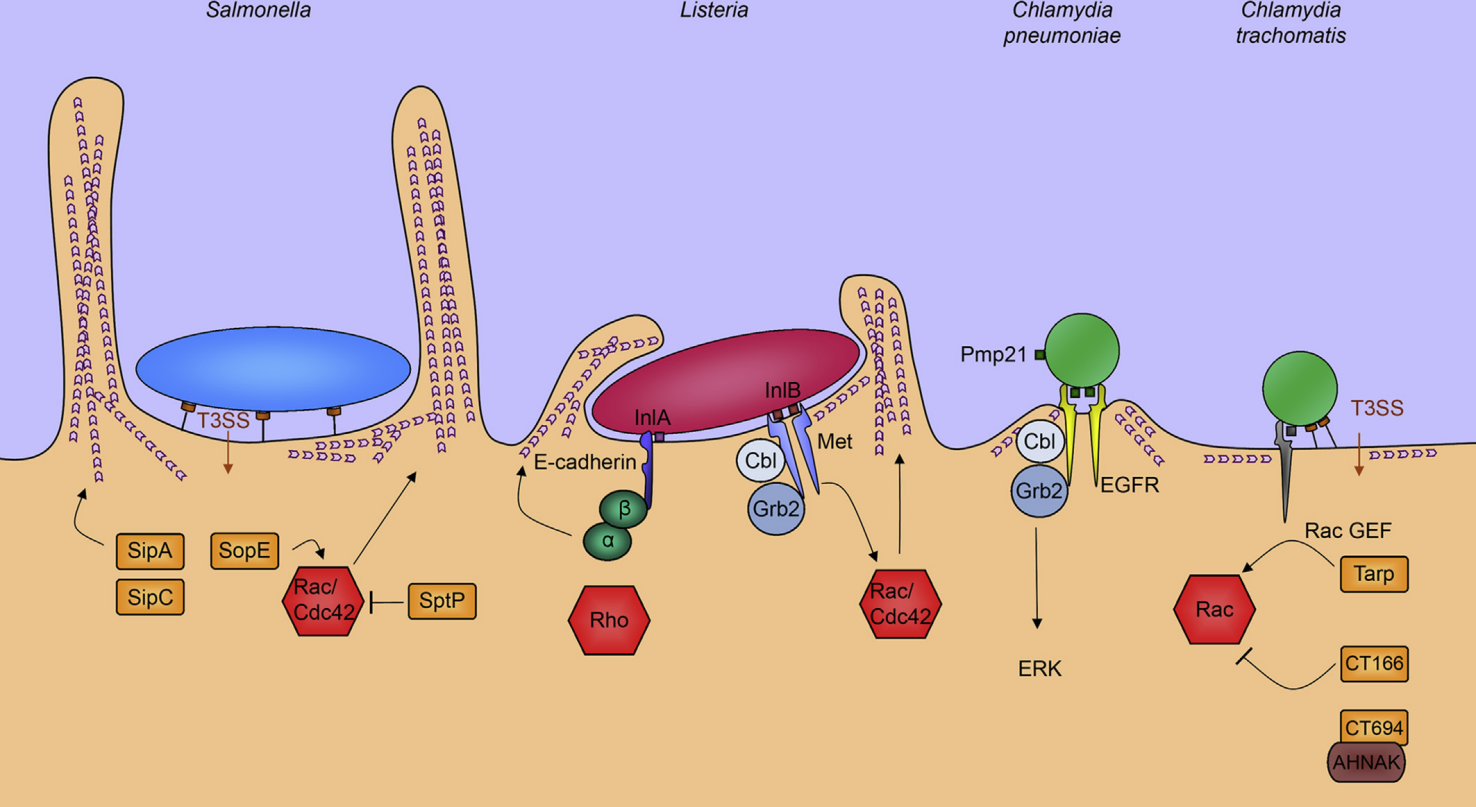
**Comparison of chlamydial entry pathways and the archetypal ‘trigger’ and ‘zipper’ mechanisms of bacterial entry**. Schematic summarising the mechanisms of cell entry by *Salmonella typhimurium*, *Listeria monocytogenes*, *Chlamydia pneumoniae* and *Chlamydia trachomatis. Salmonella* (blue) is the archetypal example of the ‘trigger’ mechanism. *Salmonellae* deliver T3SS effectors, *Salmonella* invasion proteins (Sips) and *Salmonella* outer proteins (Sops) (orange), which cooperate to induce actin reorganisation by directly binding and manipulating actin or via reversible stimulation of Rho-family GTPases Cdc42 and Rac1 (red). *Listeria monocytogenes* (red) is the archetypal example of the ‘zipper’ mechanism. *Listeria* uses surface internalins (InlA, InlB; brown) to bind cognate receptors (E-cadherin, Met; dark blue) to stimulate actin reorganisation via signalling through adaptor proteins (catenins, green; Grb2, Cbl, violet) and Rho-family GTPases (red). *Chlamydia pneumoniae* elementary bodies (green) utilise the species-specific Pmp21 surface protein to engage and stimulate signalling via epidermal growth factor receptor (EGFR, yellow) to promote bacterial entry. *Chlamydia trachomatis* elementary bodies (green) engage multiple receptors and deliver T3SS effectors including Tarp and CT166 (orange) to reversibly stimulate the Rho-family GTPase Rac1 (red) and trigger bacterial internalisation. CT694 engages host AHNAK to promote cytoskeletal reorganisation.

Cell entry by *C. trachomatis* is often considered to be an example of the ‘trigger’ mechanism of bacterial entry, epitomised by the enteroinvasive bacterium *Salmonella typhimurium* [21]. Like *Salmonella*, *C. trachomatis* delivers T3SS effectors into the host cell that reversibly stimulate the Rho-family GTPase Rac1 [22]. Understanding of this process is far from complete, but the T3SS effector Tarp nucleates actin polymerisation directly and acts as a scaffold for Rac1 guanine nucleotide exchange factors [23], [24], whereas CT166 post-translationally modifies the GTPase itself [25]. Rac1 stimulation is sufficient to induce cytoskeletal rearrangements and the formation of lamellipodia [26], although this activity alone cannot account for the diversity in chlamydial entry structures observed (Fig. 3).

A number of host cell receptors are also implicated in cell entry by different chlamydial species. *C. pneumoniae* uses the species-specific outer membrane protein Pmp21 as an invasin to bind and stimulate epidermal growth factor receptor (EGFR), and induce EB uptake by receptor-mediated endocytosis [27]. Indeed, this event is more reminiscent of the ‘zipper’ mechanism exemplified by *Listeria monocytogenes* in which a single bacterial ligand mimic is sufficient to drive bacterial entry [28]. In addition to the action of the T3SS, the role of receptors in *C. trachomatis* entry is less clear. Mannose receptor enhances *C. trachomatis* adhesion [29], the cystic fibrosis transmembrane conductance regulator (CFTR) binds chlamydial LPS [30], and most recently Ephrin A2 has been linked to *C. trachomatis* adhesion and entry, although invasion was only reduced by 30% upon receptor silencing [31]. EGFR activity is also important for the progression of the *C. trachomatis* lifecycle [32]. However, none of these receptors are essential for *C. trachomatis* entry, reinforcing the view that multiple or redundant entry pathways are likely to operate in parallel. One potential common factor is protein disulphide isomerase (PDI), which is essential for cell adhesion by *C. trachomatis*, *Chlamydia pneumoniae* and *C. psittaci*. However, PDI does not interact directly with *Chlamydia* and is instead proposed to act enzymatically as part of diverse receptor complexes, or potentially directly to activate the T3SS by targeting the needle-forming protein CdsF or reduce disulphide cross-links in outer membrane proteins [33], [34], [35]. Moreover, *C. trachomatis* invasion was reduced by 97% when both PDI and Ephrin A2 were simultaneously silenced [31].

## 5 Concluding remarks

Mounting evidence therefore suggests that *C. trachomatis* exploits facets of both the archetypal ‘trigger’ and ‘zipper’ mechanisms of bacterial entry into host cells [21]. Given the small diameter of EBs and the structures observed during entry, elements of ‘surfing’ and ‘capture’ more reminiscent of events during viral interaction with the host cell plasma membrane are also suggested. What is clear is that there is still much to learn from using a combination of structural, cellular and molecular approaches to study the critical early interactions between *C. trachomatis* and host cells.

## Conflicts of interest

The authors declare no conflicts of interest.
